# microRNA exchange via extracellular vesicles in cancer

**DOI:** 10.1111/cpr.12877

**Published:** 2020-10-06

**Authors:** Luyen Tien Vu, Jinhua Gong, Thach Tuan Pham, Yeokyeong Kim, Minh T. N. Le

**Affiliations:** ^1^ Department of Pharmacology Yong Loo Lin School of Medicine National University of Singapore Singapore; ^2^ Department of Biomedical Sciences College of Veterinary Medicine and Life Sciences City University of Hong Kong Kowloon Hong Kong; ^3^ City University of Hong Kong Shenzhen Research Institute Shenzhen China

**Keywords:** cancer biology, extracellular vesicles, microRNAs, tumour microenvironment

## Abstract

Cells utilize different means of inter‐cellular communication to function properly. Here, we review the crosstalk between cancer cells and their surrounding environment through microRNA (miRNA)‐containing extracellular vesicles (EVs). The current findings suggest that the export of miRNAs and uptake of miRNA‐containing EVs might be an active process. As post‐transcriptional regulators of gene expression, cancer‐derived miRNAs that are taken up by normal cells can change the translational profile of the recipient cell towards a transformed proteome. Stromal cells can also deliver miRNAs via EVs to cancer cells to support tumour growth and cancer progression. Therefore, gaining a better understanding of EV‐mediated inter‐cellular communication in the tumour microenvironment might lead to the development of novel diagnostic and therapeutic strategies.

## Graphical abstract

Cancer cells communicate mutually with tumour microenvironment (TME) cells via extracellular vesicles (EVs). Cancer cells secrete miRNAs into EVs that are subsequently taken up by TME cells in which miRNAs promote a tumour‐supportive phenotype. Cancer cells also receive exogenous miRNAs from TME cell–derived EVs to support tumour growth. This bidirectional signalling results in tumour progression and metastasis.

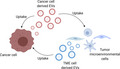

## 1 INTRODUCTION

Membrane trafficking can take place intra‐ or inter‐cellularly.^1^, ^2^ While intracellular membrane trafficking has been well‐studied, inter‐cellular communication via extracellular vesicles (EVs) has only recently emerged as a novel cell signalling mechanism.^3^ EVs are membrane‐bound nanoparticles, which are secreted by all cell types.^4^ Different types of EVs have been annotated and their classification is based on biogenesis. For example, living cells secrete both exosomes (~40‐100 nm in diameter) and ectosomes or microvesicles (~100‐1000 nm in diameter). The former originates from endosomal multivesicular bodies (MVBs) that fuse with the cell membrane, whereas the latter directly bud from the cell membrane upon stimulation. There are also apoptotic bodies (>500 nm), which are formed when cells undergo programmed cell death. Of note, the size of the EVs presented here is for reference only and cannot be utilized as a classification characteristic. In addition to the well‐known EV species mentioned above, various other types of vesicles have also been described.^5^ However, their formation might still follow the basic biogenesis steps of exosomes and microvesicles.

EVs contain a wide array of biological molecules, which represent the cytoplasmic and membranous contents of the mother cell. These include membrane proteins, enzymes, signalling molecules and RNAs including both coding and non‐coding RNAs. MicroRNAs (miRNAs) are a class of small single‐stranded non‐coding RNAs (of ~22 nt in length) that play an important role in the regulation of gene expression in eukaryotes.^6^ miRNAs and associated proteins of the RNA‐silencing complex (RISC) execute their functions mainly by binding (either precisely or imprecisely) to the 3’ untranslated region (3’UTR) of their target mRNA, which subsequently leads to translational inhibition and/or target mRNA degradation in the cytosol. In mammals, most protein‐coding genes contain at least one miRNA‐binding site.^7^ Therefore, miRNA‐mediated gene regulation is involved in many important processes including embryonic development, the cell cycle and metabolism.

The expression of miRNA is tightly regulated.^6^ After being transcribed by RNA polymerase II, miRNA primary transcripts (pri‐miRNAs), which encode one or multiple miRNAs, are further processed via 5’‐capping, splicing and polyadenylation. Pri‐miRNAs can reach several kilobases in length and they contain stem‐loop structures, which are the target excision sites for the nuclear RNase III, Drosha. After cleavage by Drosha, the hairpins (pre‐miRNAs) generated are translocated into the cytosol by exportin‐5. The pre‐miRNAs then have their terminal loop removed by Dicer, and this results in the formation of double‐stranded (ds) RNAs of ~22 bp. The ds‐miRNAs are subsequently loaded onto Argonaute (Ago) protein to form a RISC. Ago selects one strand to be a guide (miRNA) and it removes the other (passenger strand or miRNA*) in order to form a functional complex.^8^

Although miRNAs are mainly localized in the cytosol, they are also secreted in extracellular complexes such as EVs. Cell‐free miRNAs have been identified in different body fluids such as serum/plasma,^9^, ^10^ breast milk,^11^ colostrum,^12^ saliva,^13^ tears,^14^ urine,^15^ seminal fluid,^16^ amniotic fluid,^17^ pleural fluid,^18^ bronchoalveolar lavage fluid,^19^ gastric juice,^20^ peritoneal fluid^21^ and cerebrospinal fluid.^22^ Moreover, the abnormal expression of miRNAs is indicative of various pathological disorders.^23^ For example, aberrant levels of specific circulating miRNAs correlate with diseases such as cancer,^24^ diabetes,^25^ cardiovascular diseases,^26^ muscular disorders^27^ and neurodegenerative diseases,^28^ as well as toxic and drug‐induced organ damage.^29^, ^30^ For this reason, many studies have been conducted to determine how circulating miRNAs might be utilized as novel biomarkers for these diseases. Interestingly, extracellular miRNAs can be taken up by and released inside the cytosol of recipient cells, which suggests that they might play an important role in inter‐cellular communication.^3^ In this review, we will discuss the current knowledge regarding the role of EV‐associated miRNAs as mediators of the cell‐to‐cell communication that occurs in cancer.

## 2 miRNA SECRETION

Stable circulating miRNAs have been found to complex with lipids,^31^ proteins^32^ and secreted vesicles,^33^ which suggests that there is more than one mechanism of miRNA secretion. In addition to a passive leakage due to cellular injury (ie necrosis), various different pathways have been proposed to explain the secretion of miRNAs into the circulation.^34^ For example, high‐density lipoprotein (HDL) and other proteins can serve as carriers for different groups of miRNAs.^31^, ^32^ However, the mechanism by which miRNAs are selected for HDL secretion is still unknown. It is known that miR‐16, miR‐92a, miR‐122, miR‐142‐3p and miR‐150 in complex with AGO2 can all stably circulate in the blood stream.^32^ However, the miRNA secretion pathway where most is known is the export of miRNAs via EVs.

Two processes govern the presence of a specific miRNA in EVs; these are loading and sorting. Loading refers to mechanisms in which EV cargoes are recruited to the site of vesicle formation, whereas sorting is an active selection process in which some specific miRNAs are exclusively destined for secretion. These processes happen simultaneously following vesicle biogenesis.

### 2.1 miRNA secretion into exosomes

**Figure 1:**
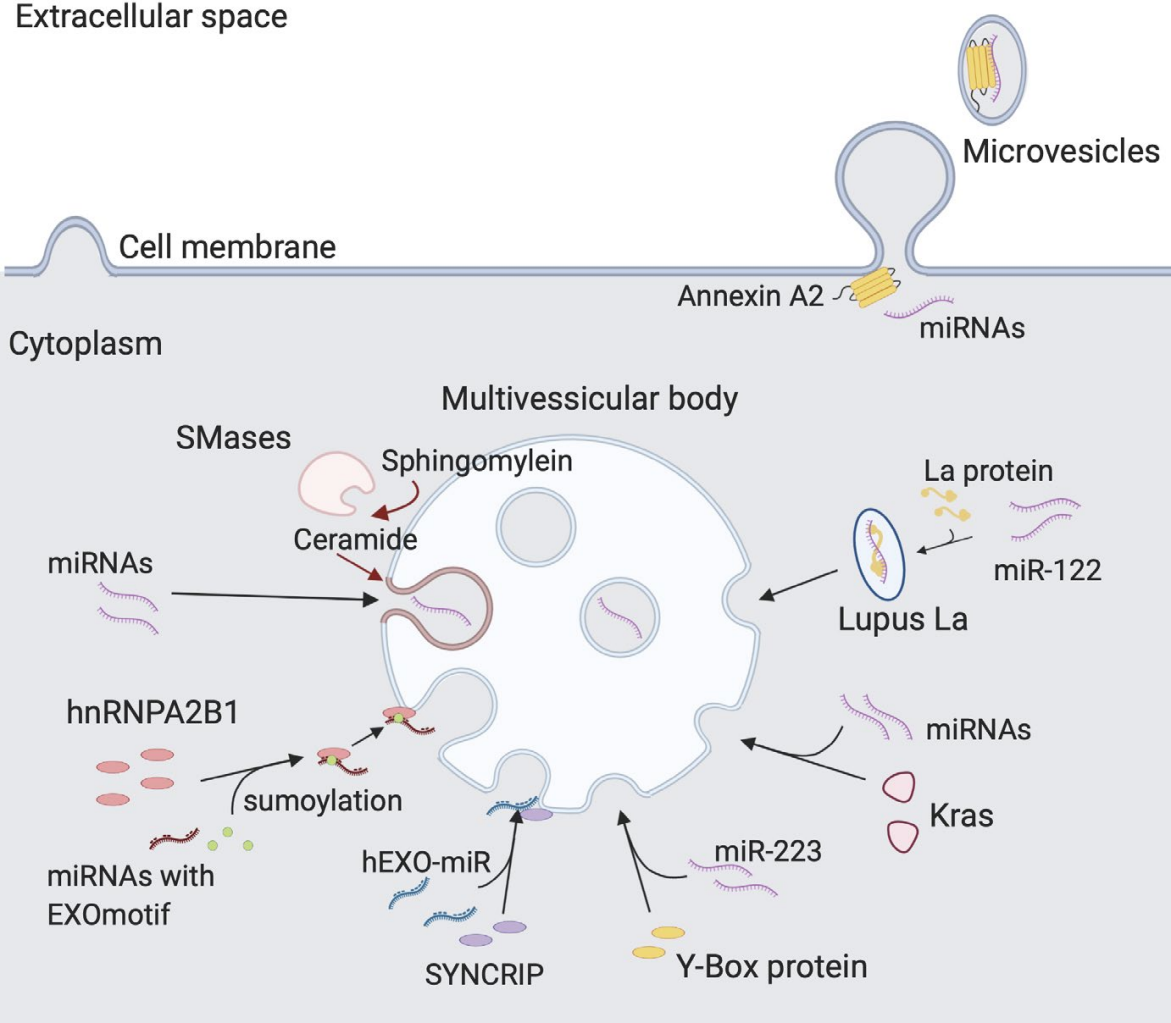
Incorporation of miRNAs into EVs. Different mechanisms govern the transfer of miRNAs from the cytosol into EVs. Several RNA‐binding proteins have been found to direct specific miRNAs to the formation sites of the ILVs on the endosome and the microvesicles on the cell membrane. EVs, extracellular vesicles; ILVs, intraluminal vesicles

Various attempts have been made to analyse the loading and sorting of miRNA into exosomes. Exosome biogenesis includes either the recruitment of the endosomal sorting complex required for transport system (ESCRT‐dependent) or the synthesis of ceramide (ESCRT‐independent).^4^ miRNA loading is more likely to be involved in the ESCRT‐independent pathway. This is because downregulation of neutral sphingomyelinase 2 (nSMase2), a key regulator of ceramide synthesis, leads to decreased miRNA secretion, whereas blocking the ESCRT has little effect on miRNA secretion (Figure 1).^35^

Some miRNAs are known to be selectively sorted into EVs, although the exact mechanisms are mostly unknown. Villarroya‐Beltri et al^36^ reported that in T cells, sumoylated heterogeneous nuclear ribonucleoprotein A2B1 (hnRNPA2B1) binds to a particular motif of various miRNAs and localizes them into the intraluminal vesicles (ILVs) of MVBs (Figure 1). These MVBs subsequently fuse with the cell membrane and release the now‐mature ILVs or exosomes. Santangelo et al^37^ reported that in hepatocytes, the RNA‐binding protein, SYNCRIP, selectively sorts miRNAs that have a 4‐nucleotide motif near the 3’ end, independently of hnRNPA2B1 (Figure 1). Wild‐type and mutant KRAS regulate the secretion of different miRNAs into colorectal cancer exosomes (Figure 1).^38^ In addition, the RNA‐binding protein called Y‐box protein 1 has been shown to selectively sort miR‐223 into exosomes in HEK‐293T cells (Figure 1).^39^ Furthermore, Lupus La protein binds to miR‐122 at both ends, to target this miRNA for secretion in exosomes of human triple‐negative breast cancer MD‐MBA231 cells (Figure 1).^40^ Together, these findings suggest that there is more than one mechanism for miRNA sorting. However, it is unclear whether the loading of miRNAs is sequence specific although some findings have suggested that the sorting of selective miRNAs is based on motif recognition.

Genomic position might also play a role in miRNA secretion. miRNAs often cluster together in the genome and are often transcribed together. By comparing the miRNAs of the cells and exosomes of related individuals, Tsang et al^41^ identified an active region at band 14q32 on chromosome 14 for the secretion of miRNAs. However, the group also stated that the selective export miRNAs could not be explained by current mechanisms.

As miRNA can be selectively sorted into EVs, the concentration of miRNA in EVs (a quantitative aspect of miRNA sorting) can provide new information regarding miRNA secretion. Squadrito et al^42^ reported that the level of miR‐511 in bone marrow–derived macrophage exosomes is affected either by the cell activation state or the expression of cellular miRNAs and their target sequences. Similarly, the expression of Cavin‐1 in prostate cancer cells was reported to selectively decrease the concentration of miR‐148a in EVs by ~3.67‐fold without changing the concentration of miR‐148a.^43^ These findings reveal that the presence and quantity of EV‐derived miRNAs are dynamic and dependent on the response of the mother cell to both internal and external signals. However, much work is still required to uncover the biological implications of the fluctuation of EV‐derived miRNAs.

### 2.2 miRNA secretion into microvesicles

Not much is known about the loading and sorting of miRNAs into microvesicles. Although the biogenesis of microvesicles and exosomes is regulated by overlapping sets of molecules, the former still utilizes a distinct collection of regulatory proteins.^44^ In the biogenesis of microvesicles, annexin A1 and A2, which are calcium ion‐ (Ca^2+^‐) and membrane‐binding proteins, play an important role in forming a curved structure on the membrane for blebbing and folding.^45^ Indeed, using a combination of high‐resolution density gradient fractionation and immunoaffinity methods to purify and classify EVs, Jeppesen et al^46^ identified annexin A1 and A2 as exclusive markers of microvesicles. Of note, these members of the annexin family are also RNA‐binding proteins.

Annexin A2 is also reported to regulate the levels of miRNAs in EVs (Figure 1).^47^ It both facilitates the loading of miRNAs into EVs and also selectively sorts a number of miRNAs, including miR‐16, miR‐21, miR‐24, miR‐29a, miR‐100, miR‐125, let‐7a and let‐7b. Recent findings show that a post‐translational isoform of annexin A2 partially colocalizes with processing (P‐) bodies where the RISC machineries are recruited.^48^ This might be a clue for the functional delivery of EV‐associated miRNAs, such that annexin A2 is modified and targets miRNAs to the P‐bodies in recipient cells.

## 3 UPTAKE OF miRNA‐CONTAINING EVs

Upon secretion, EVs are taken up by their parental cells or by other (either adjacent or distant) cells via autocrine or paracrine signalling.^49^ Although much progress has been made to elucidate the mechanisms of miRNA uptake by EVs and other carriers, the EV uptake mechanism is still not fully understood. Since studying exosomes and microvesicles separately is complicated, we will continue to use the general term “EVs” for the following section.

### 3.1 Uptake of EVs by recipient cells

**Figure 2:**
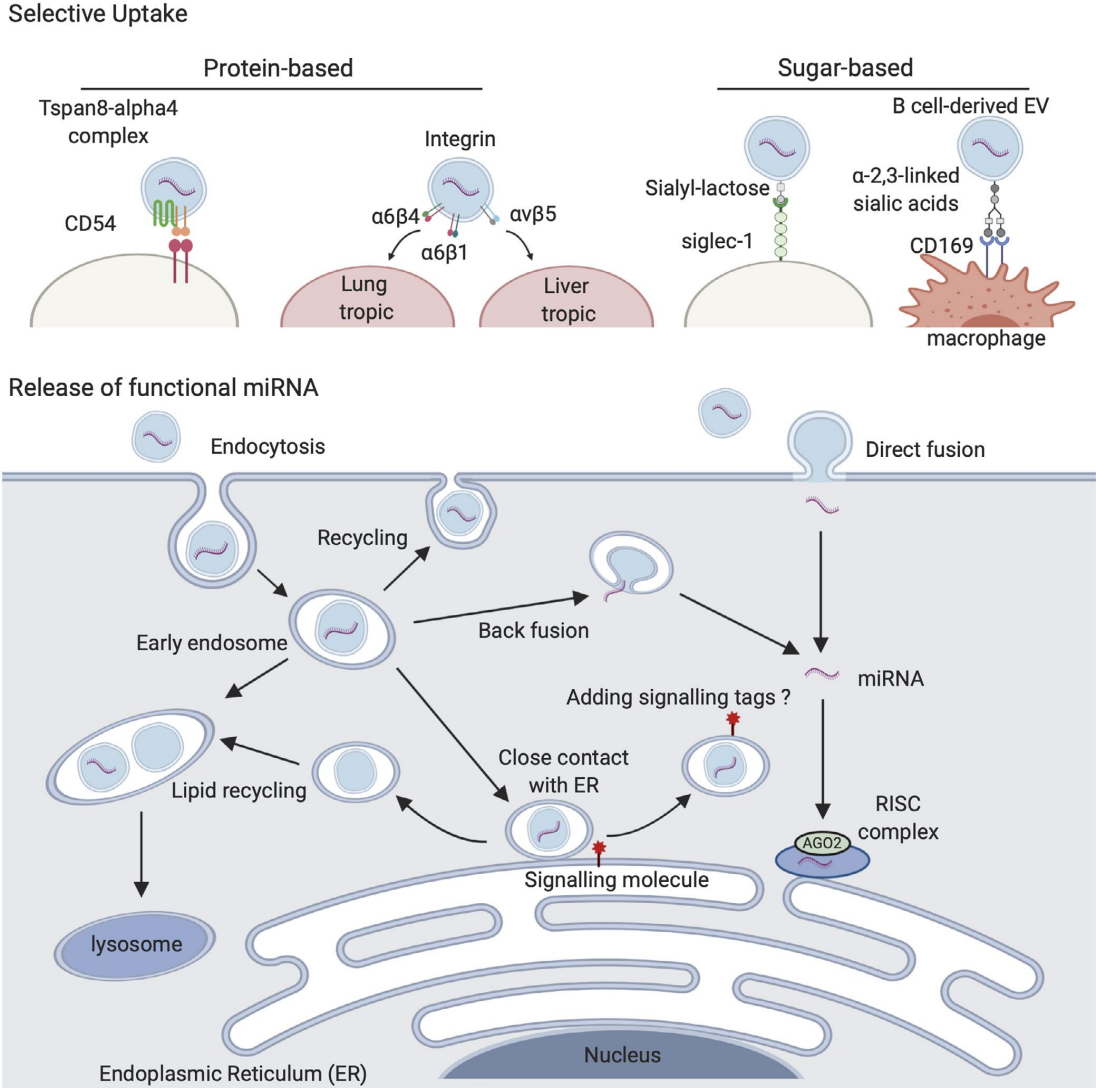
EV uptake and release of miRNAs into the cytosol of recipient cell. Proteins and sugar chains on EV surface can direct the selective binding of EVs to a specific cell. EV‐derived miRNAs are released into the cytosol by either direct fusion of the EVs to the cell membrane or the endosomal‐limiting membrane or different mechanisms. EVs, extracellular vesicles

Since EVs are carriers of proteins and other polynucleotides as well as miRNAs, the endocytic pathway is likely to be common for all EVs, whatever they carry. EVs are taken up into cells by a number of different mechanisms, including endocytosis, phagocytosis, macropinocytosis, and plasma membrane fusion, all of which utilize different sets of proteins for binding and internalization.^50^, ^51^ Therefore, it is likely that the uptake of EVs is dependent on surface proteins on the recipient cells as well as ligands on the EVs (Figure 2). Indeed, several studies have demonstrated the differential uptake of EVs by different types of recipient cells. For example, it has been shown that exosomes expressing the Tspan8‐alpha4 complex on their surface most likely bind to CD54 on the membrane of endothelial and pancreatic cells (Figure 2).^52^ Interestingly, the integrin signatures on the surface of exosomes have been shown to determine metastatic organotropism in different organs (Figure 2).^53^ For example, using labelled exosomes, Hoshino et al^53^ discovered a novel homing pattern of tumour EVs with different combinations of integrin molecules. Specifically, integrin α6β4‐ and α6β1‐positive exosomes were shown to be endocytosed more by lung epithelial cells, whereas αvβ5‐positive exosomes are more liver‐tropic. In another study, activated T cells were reported to capture major histocompatibility complex (MHC) class II–containing exosomes secreted by dendritic cells.^54^ This process was shown to be regulated in a dose‐dependent manner by leucocyte function‐associated antigen‐1 on the EV surface.^54^

Glycosylation also plays a role in the specific uptake of EVs. For example, the uptake of EVs by recipient cells is, in some cases, regulated by D‐mannose, D‐glucosamine and β‐galactosides, and the underlying mechanism involves sugar‐binding proteins.^55^, ^56^ It has been shown that sialyl‐lactose–containing EVs, which are secreted by Jurkat cells, bind to (and are captured by) dendritic cells via sialic acid‐binding Ig‐like lectin 1 (Siglec‐1) on the latter (Figure 2).^57^ In addition, Saunderson et al^58^ reported that B cell–derived EVs are taken up by lymph node macrophages due to the binding of α2,3‐linked sialic acids on the former to the sugar‐binding protein, sialo‐adhesin (CD169), on the latter (Figure 2).^59^ These findings confirm the hypothesis that the uptake of EVs is neither random nor passive, but rather is regulated by a complex series of pathways. Of note, the specificity of EV uptake leads to another question, which is are different sets of miRNAs selectively secreted into different populations of EVs with specific uptake tropism?

### 3.2 Release of EV‐derived miRNAs into the cytosol

After internalization into the cell, most EVs remain in endosomes. It is therefore challenging for EV‐derived miRNAs to get into the cytosol so that they can be loaded onto RISCs and perform their physiological role. Montecalvo et al^58^ proposed a functional transfer of miRNAs by direct fusion with the plasma membrane, but also reported that EV cargoes are released to the cytoplasm by back fusion of EVs to the endosomal‐limiting membrane (Figure 2).^58^ Direct fusion with the plasma membrane is also reported by other groups via a process that is pH dependent.^60^ However, the detailed mechanism for EV‐plasma membrane fusion is not well understood, although it has been suggested that common intracellular vesicle fusion protein families such as SNAREs, Rab proteins and Sec1/Munc‐18 might play a role.^61^

Little is known about the fate of EVs after being endocytosed. However, Heusermann et al^62^ traced the uptake of CD63+ exosomes and found that they surf on filopodia to enter recipient cells and shuffle in cytoplasm endosomes.^62^ Once these labelled exosomes enter a cell, they follow the usual endosomal trafficking routes that lead to degradation in lysosomes (Figure 2). Interestingly, around 90% of the labelled vesicles move into close association with the endoplasmic reticulum (ER) before sorted into lysosomes.^62^ In human primary fibroblasts, within 48 hours of internalization, 60% of the labelled exosomes are colocalized with lysosomes.^62^ The research group proposed the hypothesis that EVs are in contact with ER for efficient release of RNA contents near the local translation machineries.^62^ However, when EVs are labelled with a generic lipid dye or the membrane proteins are labelled with a fluorescent tag, the release of EV cargo is rarely observed.^63^ Therefore, labelling both the EV contents and membrane, and tracing the end point of EV uptake are all essential for determining the fate of EVs in the cell. Once this mechanism has been revealed, the fate of EVs might be controlled and manipulated for therapeutic purposes.

### 3.3 Loading of EV‐derived miRNAs into RISC of recipient cells

The functionality of EV‐derived miRNAs in recipient cells is another key question in the field. In order to perform translational silencing, single‐stranded miRNAs must bind to AGO2 and other proteins to form the RISC complex during their maturation.^6^ It is unclear whether single‐stranded free miRNAs released from EVs are active in recipient cells since they might interact less efficiently with AGO2, when compared with pre‐miRNAs.^64^

Interestingly, annexin A1 is also capable of binding to both DNA and RNA upon formation of an A1‐S100 heterotetramer complex; this suggests an additional possible role for annexin A1 in regulating RNA sorting in EVs.^65^

In an attempt to elucidate the underlying mechanism, Melo et al^66^ reported that the miRNAs carried in exosomes undergo maturation en route to the recipient cells as pre‐miRNAs, Dicer, AGO2 and TRBP are packaged altogether in the EVs. With this cell‐independent maturation, miRNAs are thus readily functional upon their release to the cytosol. However, this mechanism has not been confirmed as some other studies could not show the ubiquitous presence of Dicer and AGO2 in EVs when purified using gradient centrifugation.^39^, ^40^, ^46^ It has also been suggested that in some cases, mature miRNAs might change the phenotype of their recipient cells without being pre‐incorporated onto the RISC complex.^67^ Moreover, the selective sorting of miRNAs into exosomes often results in them forming complexes with proteins other than AGO.^39^, ^40^ However, the process of switching the miRNA from its carrier protein to AGO in recipient cells is also unknown. Therefore, further investigations are needed to clarify the processing of miRNAs after their release from EVs.

## 4 miRNA EXCHANGE AS INTER‐CELLULAR COMMUNICATION MECHANISM IN CANCER

With the ability to suppress the expression of multiple target mRNAs, miRNAs transferred via EVs support tumour growth at each stage of cancer progression. This has become a robust means of communication between cancer cells and the tumour microenvironment (TME). Here, we summarize our current understanding of the mutual communication both among cancer cells and between cancer cells and other cells of the TME.

### 4.1 The effects of miRNA‐containing EVs from cancer cells in the TME

**Figure 3:**
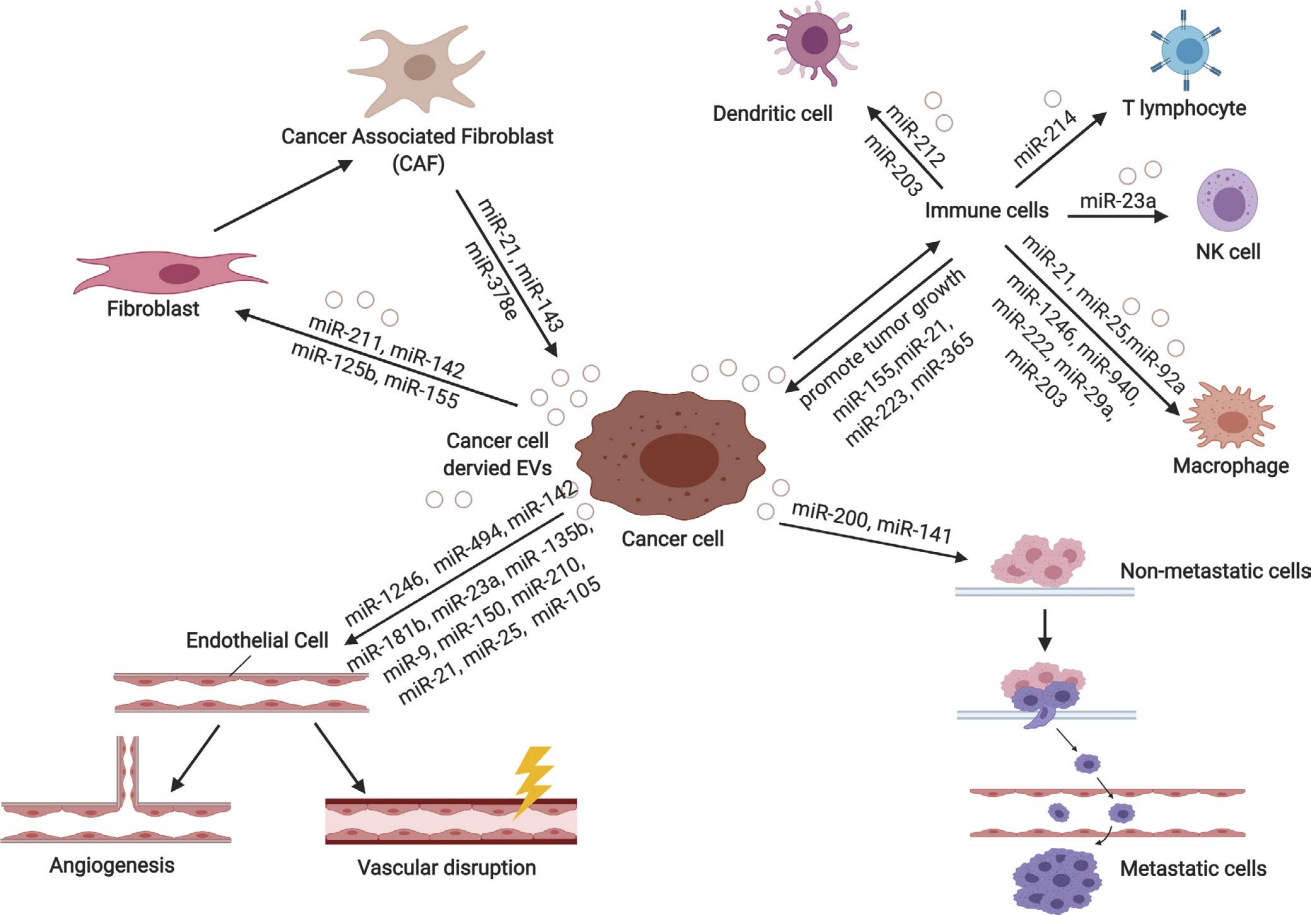
The roles of EV‐delivered miRNAs in the TME. EV‐derived miRNAs from cancer cells mediate cells of the TME into tumour supportive phenotypes. Transformed cells of the TME, in return, secrete EVs containing miRNAs that support tumour growth and progression. EVs, extracellular vesicles; TME, tumour microenvironment

Fibroblast activation is a key event in the growth of primary tumours.^68^ Our recent study revealed that breast cancer–derived EVs contain high levels of miR‐125b, which is taken up by fibroblasts situated adjacent to the primary tumour (Figure 3).^69^ This cellular increase in miR‐125b suppresses the expression of *TP53* and *TP53INP1,* and the fibroblasts differentiate into cancer‐associated fibroblasts (CAFs). A similar mechanism is observed in pancreatic cancer–derived microvesicles, which contain an elevated level of miR‐155 (Figure 3).^70^ Similarly, melanoma‐derived exosomes (melanosomes) containing miR‐211 are able to promote the formation of CAFs by activating the mitogen‐activated protein kinase signalling pathway (Figure 3).^71^ Moreover, lung adenocarcinoma cancer–derived exosomal miR‐142 has been shown to promote the differentiation of WI‐38 and IMR90 lung fibroblasts into CAFs via a TGF‐β‐independent pathway (Figure 3).^72^

Evading the immune system is a hallmark of cancer.^73^ Cancer‐derived exosomal miRNAs have been shown to regulate and suppress immune cells in order to support cancer growth. For example, Mutp53 colon cancer cell–derived exosomal miR‐1246 reprograms M2 macrophages into tumour‐associated macrophages (TAMs) (Figure 3).^74^ As a consequence, TAMs produce IL‐10, TGF‐β and matrix metalloproteinases (MMPs) to enhance tumour growth and progression. Similarly, exosomal miR‐940, which is derived from hypoxic epithelial ovarian cancer (EOC) SKOV3 cells, reprogrammes U937 macrophages into the tumour promoting M2 subtype, which in turn promotes the proliferation of EOC cells (Figure 3).^75^ EOC‐derived exosomal miR‐222 has also been shown to promote M2 macrophage polarization, and it achieved this by regulating the SOCS3/STAT3 signalling pathway (Figure 3).^76^ Again, the M2 TAM‐like macrophages enhanced EOC growth.

In addition to their canonical function, miRNAs might also exert novel functions in recipient cells. For instance, miR‐21 and miR‐29a in Lewis lung carcinoma–derived exosomes promote lung tumour multiplicities in B6 mice by binding and activating Toll‐like receptor 7 (TLR7) in the surrounding immune cells, especially macrophages (Figure 3).^77^ This results in the activation of nuclear factor κB (NF‐κB) and the secretion of pro‐inflammatory cytokines TNF‐α and IL‐6, which together promote tumour growth. Similarly, liposarcoma Lipo246 exosome–derived miR‐25 and miR‐92a can induce secretion of IL‐6 in surrounding TAMs by regulating NF‐κB signalling via TLR7/8 (Figure 3).^78^

Dendritic cells also play a part in the tumour modulation of the immune system.^79^ For example, miR‐203‐containing exosomes from pancreatic cancer cells inhibit the expression of TLR4 in recipient dendritic cells, and downregulate the production of TNF‐α and IL‐12, thereby repressing the usual anti‐tumour immune response (Figure 3).^80^ Moreover, pancreatic cancer cell PANC‐1–derived exosomal miR‐212 modulates regulatory factor X‐associated protein expression (Figure 3).^81^ This leads to a decrease in the expression of MHC‐II and thus induces immune tolerance of dendritic cells.

In addition to macrophages and dendritic cells, natural killer (NK) cells are also critical effectors in the innate immune response.^82^ Berchem et al^83^ showed that tumour‐derived exosomal miR‐23a modulates the immune response by impairing NK cytotoxicity and function (Figure 3). They demonstrated that when miR‐23a and TGF‐β were delivered by microvesicles isolated from hypoxic IGR‐Heu, K562 and T1 tumour cells into MK‐92 cells, then the expression of CD107a/LAMP1 was reduced and the number of NKG2D activator surface receptors was decreased in these cells.

miRNA‐containing EVs from tumour cells also modulate and suppress T‐lymphocyte function. The delivery of miR‐214 from Lewis lung cancer cell–derived exosomes to recipient T cells was shown to significantly downregulate PTEN and promote a regulatory T cell (Treg) phenotype (Figure 3).^84^ These Tregs in turn secret IL‐10, which promotes tumour growth.

In addition to fibroblasts and cells of the immune system, miRNAs carried by EVs can also target endothelial cells. For example, miR‐9 (carried by SK23 melanoma cell–derived microvesicles) targets SOCS5 in endothelial cells (Figure 3); this leads to activation of the JAK‐STAT pathway and results in cell migration and tumour angiogenesis.^85^ Secreted miR‐150 from leukaemia THP‐1 cell–derived EVs also confers angiogenic properties on recipient human microvascular endothelial cells (HMEC‐1) via downregulation of the c‐Myb pathway (Figure 3).^86^, ^87^ In addition, in hypoxic conditions miR‐210 carried in exosomes derived from K562 leukaemia cells or 4T1 breast cancer cells promote angiogenesis in HUVECs by downregulating the receptor tyrosine kinase ligand ephrin‐A3 (Figure 3).^88^, ^89^ Angiogenesis is also promoted when exosomal miR‐210 derived from hepatoma QGY‐7703 cells is delivered into HUVECs; in this case, *SMAD4* and *STAT6* are downregulated (Figure 3).^90^ Exosomal miR‐210 derived from TIMP‐1 overexpressing cells also promotes formation of tubes in HUVECs in vitro and enhances angiogenesis in A549L‐derived lung tumour in vivo.^91^ In addition, colorectal cancer DLD‐1 cell–derived microvesicles containing miR‐1246 were shown to promote angiogenesis in HUVECs by inhibiting the expression of promyelocytic leukaemia protein and upregulating the smad 1/5/8 signalling pathway (Figure 3).^92^ Furthermore, Mao et al^93^ reported that exosomal miR‐494 from A549 non–small cell lung cancer (NSCLC) cells promotes HUVEC migration and angiogenesis by downregulating PTEN, which results in activation of the Akt/eNOS pathway (Figure 3). PTEN (and PHLPP2) is also suppressed (and angiogenesis and metastasis are induced) in vascular endothelial cells by EVs carrying miR‐181b from oesophageal squamous cell carcinoma.^94^ Another exosomal miRNA, miR‐23a (from CL1‐5 hypoxic human lung cancer cells), also enhances HUVEC tube formation by reducing the expression of prolyl hydroxylase 1 and 2 (PHD1 and PHD2), which results in increased hypoxic‐inducible factor‐1 alpha (HIF‐1α) signalling (Figure 3).^95^ HIF‐1 transcription is also upregulated and angiogenesis is promoted in HUVECs carrying exosomal miR‐135b (derived from hypoxia‐resistant multiple myeloma; HR‐MM), via suppression of the HIF‐1 inhibitor, FIH‐1 (Figure 3).^96^ Interestingly, Sruthi et al^97^ reported that HepG2‐derived exosomal miR‐23a downregulates SIRT1 in recipient endothelial cells when promoting angiogenesis (Figure 3). In addition, exosomal miR‐21 derived from cigarette smoke extract‐transformed human bronchial epithelial cells enhances angiogenesis of HUVECs by increasing the levels of VEGF (Figure 3).^98^ Furthermore, exosomal miR‐142 derived from lung adenocarcinoma, which (as mentioned above) promotes the differentiation of lung fibroblasts into CAFs, also induces tube formation in HMEC‐1 endothelial cells by targeting TGFβR1 (Figure 3).^72^

In order to undergo metastasis, cancer cells must disrupt the vascular barrier to infiltrate the circulation system.^73^ miR‐105 targets the mRNA encoding the tight junction protein ZO‐1 and in this way disrupts the vascular barrier and facilitates the metastasis of cancer cells (Figure 3).^99^

Creating a niche is another premise for metastasis. MCF10A breast cancer cells secrete EVs containing miR‐122. This leads to downregulation of the expression of the glycolytic enzyme pyruvate kinase (PKM2) and GLUT1 genes in recipient lung fibroblasts and brain astrocytes in a pre‐metastatic niche, resulting in decreased glucose uptake and metabolism in these cells and thereby promoting breast cancer metastasis.^100^ In addition, colorectal cancer–derived exosomal miR‐21 binds to TLR7 in liver macrophages (Figure 3).^101^ Through the TLR7 signalling pathway, liver macrophages polarize to a pro‐inflammatory phenotype and produce cytokines such as IL‐6, thus generating a pre‐metastatic niche for colorectal cancer in the liver. Moreover, miR‐203 containing exosomes released from colorectal cancer cells induces the differentiation of monocytes into M2 macrophages in vivo and thus promotes liver metastasis (Figure 3).^102^ Furthermore, exosomal miR‐25 from SW480 colorectal cancer cells enhances angiogenesis at pre‐metastatic niches in endothelial cells, by silencing *KLF2* and *KLF4,* which restricts the expression of tight junction related proteins such as VEGFR2, ZO‐1, occludin and claudin‐5 (Figure 3).^103^

These findings demonstrate that the transfer of miRNAs from cancer cells to cells of the TME is an active and dynamic process. The same miRNA (ie miR‐21, miR‐23a, miR‐142, miR‐1246) can have different effects on the recipient cells by targeting different mRNAs or interacting with different proteins. Of note, the miRNAs being studied here are usually the most abundance miRNAs in the cancer‐derived EVs. The role of miRNAs with low abundant in EVs is more difficult to assess. These miRNAs are usually excluded from functional analysis because their addition to EV‐recipient cells is likely insufficient to make any significant functional impact. Further studies are desirable to investigate the combinatory effects of multiple miRNAs in EVs on their recipient cells.

### 4.2 Stromal‐derived miRNAs

The TME also communicates with cancer cells via miRNAs in EVs. For example, upon activation of ovarian CAFs and cancer‐associated adipocytes, miR‐21–containing EVs are secreted, and these target apoptotic protease activating factor 1 (*APAF1*) to promote chemoresistance and suppress apoptosis of recipient cancer cells (Figure 3).^104^ Neuroblastoma cells also secrete exosomal miR‐21, which upregulates miR‐155 levels in TAM and TAM‐derived exosomes (Figure 3).^105^ The latter then carry miR‐155 to neuroblastoma cells where they downregulate telomeric repeat binding factor 1, thereby enhancing neuroblastoma chemoresistance. TAMs also carry miR‐223 to breast cancer cells and enhance cancer invasion via the Mef2c‐β‐catenin pathway (Figure 3).^106^ In addition, M2 subtype TAMs secrete exosomal miR‐21, which regulates the PTEN/PI3K/AKT signalling pathway in gastric cancer cells, thereby enhancing cisplatin resistance (Figure 3).^107^ Binenbaum et al^108^ demonstrated that TAM‐derived exosomal miR‐365 can induce both in vivo and in vitro chemoresistance in K989 pancreatic adenocarcinoma cells by upregulating triphosphate nucleotide (NTP) (Figure 3).^108^ Increased levels of NTP induce the expression of cytidine deaminase, which can metabolize the chemotherapeutic drug, gemcitabine, to its inactive form. Aggressive properties such as the stemness, endothelial‐to‐mesenchymal transition, anchorage‐independent growth and invasive capacity of T47D, BT549 and MDA‐MB‐231 breast cancer cells are all enhanced by breast CAF‐secreted exosomal miR‐21, miR‐143, and miR‐378e (Figure 3).^109^ In addition, exosomal miR‐21 secreted by hypoxic mesenchymal stem cells is reported to promote NSCLC A549 cell proliferation and mobility by inhibiting the expression of *PTEN, PDCD4 and RECK* genes.^110^ The expression of PTEN is also downregulated when brain astrocyte–derived exosomes transfer miR‐19a to melanoma B16BL6 cells, and subsequently recruit myeloid cells to promote brain metastatic tumour cell growth.^111^

### 4.3 miRNAs transfer between cancer cells

As well as modulating the surrounding cells, cancer cells can also exchange EVs to enhance their progression, especially their metastatic capability via miRNAs.^67^ For example, when poorly metastatic mouse breast cancer cells take up EVs from highly metastatic isogenic cells, they become more metastatic. The underlying mechanism involves the enrichment of miR‐200 family miRNAs exclusively in the highly metastatic breast cancer cells and their secreted EVs (Figure 3). miR‐200s target *Zeb2* to drive the recipient cells towards a mesenchymal‐to‐epithelial transition, and this facilitates the colonization of poorly metastatic cells at distant sites.^67^ The same phenomenon has also been observed in MB‐231 breast cancer cells, which exhibit enhanced lung colonization in mice models after being incubated with EVs from MCF‐10CA1a cells.

Together, these findings demonstrate that communication between cancer cells and their environment is bidirectional. Thus, cancer cells secrete miRNA‐containing EVs, which convert cells nearby into a more tumorigenic (activated) state. Meanwhile, these activated cells support the growth and progression of the tumour by transferring miRNAs into the cancer cells. These findings provide a better understanding of the crosstalk between cancer cells and their microenvironment and facilitate the development of novel therapeutic treatments and early diagnosis.

## 5 CONCLUSIONS AND FUTURE PERSPECTIVES

Further investigations are still required to fully understand the crosstalk between cancer cells and their microenvironment via EVs. Among the various cargos (ie proteins and other RNAs), miRNAs play an important role as signalling molecules. However, not enough research has been conducted to determine the synergistic contribution of different molecules on the signal transduction between cancer cells and the microenvironment. As RNA‐profiling, proteome analysis and bioinformatics are advancing at a rapid rate, the role of EV‐miRNAs can be elucidated further through a comparison of the EV contents and network mapping of the genome and proteome.

Additional problems in the field include both the nomenclature and the purification of EVs.^112^ Currently, EVs are classified by their biogenesis. However, experiments often rely on the size and a handful of surface molecules to distinguish EVs. Different publications have shown that the use of such markers cannot clearly distinguish between different types of EVs.^46^ Thus, it is necessary to look for novel markers or combinations of molecules that clearly identify the diverse reservoir of EVs released by cancer cells. Identification of such markers will also facilitate the purification of EVs, as currently this also varies widely between publications.

Different mechanisms might govern the export of miRNAs. However, it is still unclear which pathways contribute the most to the level of circulating miRNAs for each cancer type. Elucidating the export mechanism of miRNAs would help to support their application as biomarkers for cancer. This would be highly advantageous as the detection of miRNAs is both fast and robust, and thus, they would be a valuable tool for cancer diagnosis and prognosis.

## AUTHOR CONTRIBUTION

LTV, GJ and TTP prepared the manuscript. TTP and YK provided illustrations. MTL obtained funding, trained and wrote the manuscript. The authors declare no competing interests.

## Data Availability

Data sharing is not applicable to this article as no new data were created or analysed in this study.
